# Electroacupuncture Reduces Aβ Production and BACE1 Expression in SAMP8 Mice

**DOI:** 10.3389/fnagi.2015.00148

**Published:** 2015-07-29

**Authors:** Wei-Guo Dong, Feng Wang, Ye Chen, Xue-Hua Zheng, Yong-Cai Xie, Wan-Qing Guo, Hong Shi

**Affiliations:** ^1^Department of Integrated Traditional Chinese and Western Medicine, Fujian University of Traditional Chinese Medicine, Fuzhou, China; ^2^The Third People’s Hospital of Fujian Province, Fuzhou, China

**Keywords:** Alzheimer’s disease, electroacupuncture, memory impairment, BACE1, amyloid-β

## Abstract

Electroacupuncture (EA) has been reported to have beneficial effects on Alzheimer’s disease (AD). BACE1 (β-site amyloid precursor protein-cleaving enzyme 1) is involved in the abnormal production of amyloid-β plaque (Aβ), a hallmark of AD pathophysiology. Thus, the present study investigated the effects of EA on memory impairment, Aβ production, and BACE1 expression in senescence-accelerated mouse prone 8 (SAMP8) mice. We found that EA improved spatial learning and memory impairment of SAMP8 mice. Furthermore, EA attenuated Aβ production and repressed the expression of BACE1 in the hippocampus of SAMP8 mice. Taken together, our results suggest that EA could have a potential therapeutic application in AD and that BACE1 may be an important target of EA in the treatment of AD.

## Introduction

Alzheimer’s disease (AD) is a progressive neurodegenerative disease characterized by two neuropathological hallmarks: extracellular senile plaque deposits, composed of amyloid β-peptide (Aβ), and intracellular neurofibrillary tangles. Ample evidence indicates that Aβ plays a central role in AD pathogenesis (Hardy and Selkoe, 2002; Selkoe and Schenk, 2003; Zhang and Xu, 2007). Aβ is generated after the cleavage of amyloid precursor protein (APP) by the β-secretase [β-site amyloid precursor protein-cleaving enzyme 1 (BACE1), β-site APP cleaving enzyme 1], and γ-secretase complex (Hardy and Selkoe, 2002). In addition, BACE1 expression and activity levels are increased in AD brains and correlate with specific regions affected by Aβ deposition (Fukumoto et al., 2002; Holsinger et al., 2002; Li et al., 2004; Harada et al., 2006; Coulson et al., 2010). Targeting BACE1 with small interfering RNA (siRNA) and BACE1 gene deletion reduces Aβ levels and prevents learning and memory impairment in a mouse model of AD (Ohno et al., 2004; Singer et al., 2005). Thus, BACE1 is considered a promising target for prevention or modification of AD (Li et al., 2006; Menting and Claassen, 2014). Moreover, several BACE1 inhibitors have entered clinical trials (Yan and Vassar, 2014).

The senescence-accelerated mouse prone 8 (SAMP8) strain is a spontaneous animal model of accelerated aging. SAMP8 mice exhibit cognitive and behavioral alterations and neuropathological abnormalities observed in patients with AD, such as deficits in learning and memory, Aβ deposits, and tau phosphorylation (Pallas et al., 2008; Takeda, 2009; Manich et al., 2011). Thus, SAMP8 mice have been proposed as an excellent model for exploring the pathophysiological mechanism of sporadic AD and a plausible experimental model for developing preventative and therapeutic treatments for late-onset/age-related AD, which accounts for the vast majority of cases in humans (Cheng et al., 2014; Bayod et al., 2015). Senescence-accelerated resistant mouse 1 (SAMR1), which has a genetic background similar to that of SAMP8, does not exhibit these senescence-related neuronal phenotypes and has been used extensively as a control strain (Takeda, 1999, 2009). In addition, BACE1 protein levels and activity were increased in SAMP8 mice compared with age-matched SAMR1 mice (Zhou et al., 2012; Orejana et al., 2015).

Acupuncture penetrates the skin with needles and stimulates specific points on the body. It has long been used for the treatment of many diseases. Electroacupuncture (EA) is a reformative form of traditional acupuncture that is accepted by Western countries due to its reproducibility (Chon and Lee, 2013). EA or acupuncture has been shown to improve cognitive deficits in animal models of AD (Cheng et al., 2008; Li et al., 2012, 2014; Wang et al., 2014a). Moreover, acupuncture activates specific cognitive-related regions and enhances hippocampal connectivity in AD patients (Wang et al., 2012, 2014c). Our previous studies demonstrated that EA at Dazhui (GV14) and Shenshu (BL23) in SAMP8 mice improves cognitive deficits, activates AMP-activated protein kinase (AMPK), and upregulates Sirtuin 1 (SIRT1)-dependent peroxisome proliferator- activated receptor-γ co-activator-1α (PGC-1α) expression (Dong et al., 2015). A recent study showed that the SIRT1-PPARγ-PGC-1 pathway may regulate BACE1 expression under metabolic stress conditions (Wang et al., 2013). Both activation of the SIRT1 signaling pathway and PGC-1α overexpression attenuate BACE1 expression and Aβ levels (Katsouri et al., 2011; Marwarha et al., 2014). On the basis of these studies, we speculate that EA may reduce the expression BACE1 and consequently reduces Aβ levels.

In this study, we evaluated the effects of EA on spatial learning and memory impairment in SAMP8 mice. Furthermore, we examined the effects of EA on Aβ production and BACE1 expression.

## Materials and Methods

### Animals and groups

This study utilized 32 male SAMP8 mice and 16 male homologous SAMR1 mice (7 months of age), which are provided by the Department of Laboratory Animal Science of Peking University. There was no difference between their baseline ages. All mice were housed under standard conditions at 22 ± 2°C and a 12 h light/dark cycle with free access to food and water throughout the entire study. The mice were randomly assigned to the following three groups (*n* = 16/group): SAMR1 normal control group (Rc), SAMP8 control group (Pc), and SAMP8 EA group (Pe). Mice in the Pe group received EA administration, while mice in the Rc and Pc groups were grasped for the same amount of time and with the same extent of strength as mice in the Pe group. Moreover, mice in the Rc and Pc groups were restrained in nets for the same length of time as mice in the Pe group. All experimental procedures were approved by the Ethical Committee of Fujian University of Traditional Chinese Medicine and were performed according to the internationally accepted principles for laboratory animal use and care. We made all efforts to minimize the animal suffering in the study.

### EA treatment

Electroacupuncture treatment was performed as described in our previous study (Dong et al., 2015). Briefly, we used nets to fix the mice by the assistant’s hands during the entire treatment. Three stainless steel acupuncture needles of 0.25 mm in diameter were inserted at a depth of approximately 5 mm into the “Dazhui” acupoint (GV14, below the spinous process of the seventh cervical vertebra) and the bilateral “Shenshu” acupoint (BL23, at the depression lateral to the lower border of the spinous process of the second lumbar vertebra). The acupoint location in the mouse is mainly in accordance with the transpositional method, which locates the animal acupoints on the surface of the animal’s skin, corresponding to the anatomical site of the human acupoint. Thus, the anatomical site and location of Dazhui in both humans and mice are the same, and are located below the spinous process of the seventh cervical vertebra. Shenshu in humans is located 1.5 cun lateral to the lower border of the spinous process of the second lumbar vertebra. On the basis of the transpositional method, Shenshu in a mouse is located at the depression lateral to the lower border of the spinous process of the second lumbar vertebra. Needles at GV14 and the side BL23 were connected to the output terminals of the EA instrument (Hwato, model No. SDZ-V, Suzhou Medical Instruments Co., Ltd., Suzhou, China) and bilateral BL23 was alternated. We performed continuous-wave stimulation at a frequency of 2 Hz (intensity 1 mA). An individual EA session was administered daily for 20 min, for 8 days, with 2 days of rest, for a period of 30 days. Each treatment for all the mice has been performed at the same day and the same time point during EA treatment.

### Morris water maze test

At the end of the 30 days of EA treatment, mice were tested for spatial learning and memory in the Morris water maze (MWM). The apparatus consisted of a large circle tank (120 cm in diameter, 30 cm in height) and was filled with opaque water (21–23°C) with white milk. The pool was divided into four quadrants of equal area. A platform (10 cm in diameter) was placed 1 cm below the water surface at the midpoint of one of the four quadrants. The position of the platform was unchanged during the training session. Before the acquisition training session, a visible platform test was performed, which confirmed that there were no significant differences in sensory, motor, or motivational activities among these three groups. Next, a hidden platform test was performed in succession. The paradigm consisted of four trials per day over five consecutive days in which the mice were placed at one of four different start positions for each trial and given 90 s to locate the hidden platform. In each trial, the escape latency was defined as the time a mouse spent from being placed into the water to finding and climbing onto the hidden platform. If a mouse failed to locate the platform within 90 s, then it was gently guided onto the platform and allowed to remain there for 10 s, and its escape latency was recorded as 90 s. Retention memory was evaluated on probe trials presented on day 6. During the probe trial, the platform was removed from the pool. The mice were released into the water on the opposite side of the target quadrant and were allowed to swim freely for 60 s. The total number of times that each mouse crossed the position where the platform was once placed and the time that it spent in the target quadrant and opposite target were recorded.

### Tissue collection

At the end of the MWM test, mice were anesthetized intraperitoneally (i.p.) with 10% chloral hydrate (100 g/0.35 mL) and immediately perfused transcardially with 50 mL of phosphate-buffered saline (PBS). The brains were immediately removed and the hippocampi were then isolated, frozen on powdered dry ice, and stored at −80°C until further assay.

### Western blotting analysis

Frozen hippocampi were homogenized in RIPA buffer supplemented with protease inhibitor cocktail and centrifuged at 12,000 × *g* for 20 min at 4°C. The supernatants were collected, and total protein levels were quantified using a BCA protein assay kit (Beyotime, Haimen, Jiangsu, China). Equal amounts (30 μg) of each sample were separated on 10% SDS-polyacrylamide gels. After electrophoresis, the proteins were transferred onto a nitrocellulose membrane (Millipore) at 100 V for 60 min on ice. The membrane was blocked with 5% w/v non-fat dry milk powder in Tris-buffered saline with 0.05% Tween 20 (TBS-T) for 1 h. After blocking, membranes were incubated with the primary antibodies overnight at 4°C. The following primary antibodies were used: anti-Aβ (6E10, 1:1000, Covance), rabbit polyclonal anti-BACE1 (1:1000, Sigma), and anti-β-actin (1:5000, Sigma). Immunoreactive bands were detected with HRP-conjugated goat anti-rabbit IgG (1:2000, Santa Cruz Biotechnology). The membrane was washed with TBS-T and the immunocomplex was visualized using an enhanced chemiluminescence detection kit (Thermo Scientific, Rockford, IL, USA). Signals of the membrane were scanned using the FluorChem Scanner and quantified using NIH Image J software. These results were normalized against β-actin expression levels and confirmed by triplicate measurements of the same sample.

### Determination of Aβ levels by enzyme-linked immunosorbent assay

For human Aβ1-42 levels detection, frozen hippocampal samples were homogenized in 5 M guanidine hydrochloride, and centrifuged at 16,000 × *g* for 20 min at 4°C. The supernatant was collected and diluted with dilution buffer plus protease inhibitor cocktail. Levels of Aβ1-42 were measured using a human Aβ1-42 Enzyme-linked immunosorbent assay (ELISA) kit (Invitrogen) according to the manufacturer’s instructions. The absorbance was read at 450 nm using a 96-well plate reader, and Aβ1-42 levels were calculated from a standard curve and normalized against total protein levels, as determined using the BCA protein assay kit.

### Quantitative real-time reverse transcription polymerase chain reaction

Total RNA was isolated using the RNeasy Mini Kit (Qiagen). Next, cDNA was synthesized using the TaqMan reverse transcription reagents kit (Applied Biosystems) and real-time PCR was performed on a 7300 real-time PCR system (Applied Biosystems, CA, USA) using the SYBR Green PCR master mix (Applied Biosystems) according to the manufacturer’s instructions. The primers used for real-time PCR were the following: BACE1 forward: 5′-CCGGCGGGAGTGGTATTATGAAGT-3′, reverse: 5′-GATGGTGATGCGGAAGGACTGATT-3′; GAPDH forward: 5′-TGGAAAGCT GTGGCGTGAT-3′, reverse: 5′-TGCTTCACCACCTTCTTGAT-3′. The data were analyzed according to the delta–delta Ct (ΔΔCT) method and were normalized against GAPDH expression in each sample.

### Statistical analysis

The results are expressed as the mean ± SEM. The escape latency of mice in the MWM test was analyzed using two-way analysis of variance (ANOVA) for repeated measurement. Tukey’s test was further used as a *post hoc* test to detect between-group differences. One-way ANOVA was employed to analyze other data obtained in these experiments followed by LSD (equal variances assumed) or Dunnett’s T3 (equal variances not assumed) for the *post hoc* test between groups. *P* < 0.05 was considered statistically significant.

## Results

### Effect of EA on memory impairment in SAMP8 mice in the MWM test

**Figure 1:**
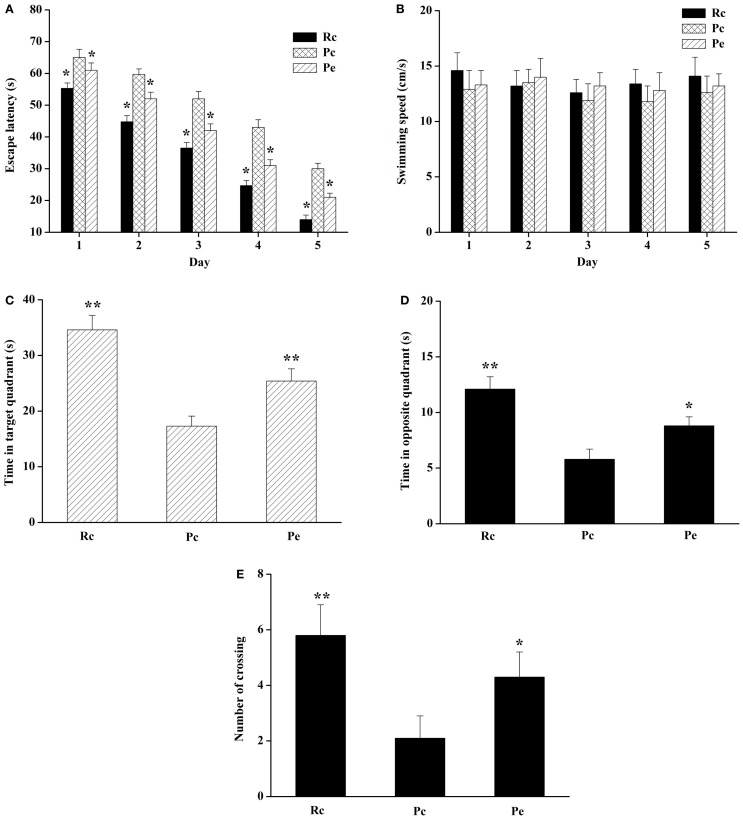
**Effects of EA on spatial learning and memory impairment in SAMP8 mice**. **(A)** Effect of EA on the escape latency of mice during five consecutive days of the hidden platform test. Pc mice exhibited a longer escape latency in the training session compared to Rc mice. EA significantly reduced the escape latency in SAMP8 mice. **(B)** Histograms showing the swimming speed during acquisition training. **(C)** Histograms showing the average swim time in the target quadrant during the probe test. **(D)** Histograms showing the average swim time in the opposite quadrant. **(E)** Comparisons of the number of platform crossings in the probe trial. Data are represented as the mean ± SEM (*n* = 14–16). *P < 0.05, **P < 0.01 versus Pc.

The MWM test was performed to detect the effect of EA on spatial learning and memory ability. The escape latency during acquisition training is shown in Figure 1A. The escape latency in the hidden platform acquisition phase decreased with an increase in the training day. Compared to Rc mice, Pc mice exhibited significantly longer escape latencies in the training session. EA significantly shortened the escape latency in SAMP8 mice. However, swimming speed was not significantly different among these groups (Figure 1B). After the training test, the probe test was performed to analyze the maintenance of memory. Pc mice exhibited a trend toward decreased time spent in the target quadrant and opposite quadrant compared with Rc mice, and EA increased the time spent by SAMP8 mice in the target quadrant and opposite quadrant (Figures 1C,D). In addition, the number of platform crossings was significantly reduced in Pc mice compared with Rc mice, and EA increased the number of platform crossings in SAMP8 mice (Figure 1E). Taken together, these results indicated that spatial learning and memory were impaired in SAMP8 mice and that EA could improve this cognitive impairment.

### EA treatment inhibits Aβ production in the SAMP8 mouse hippocampus

**Figure 2:**
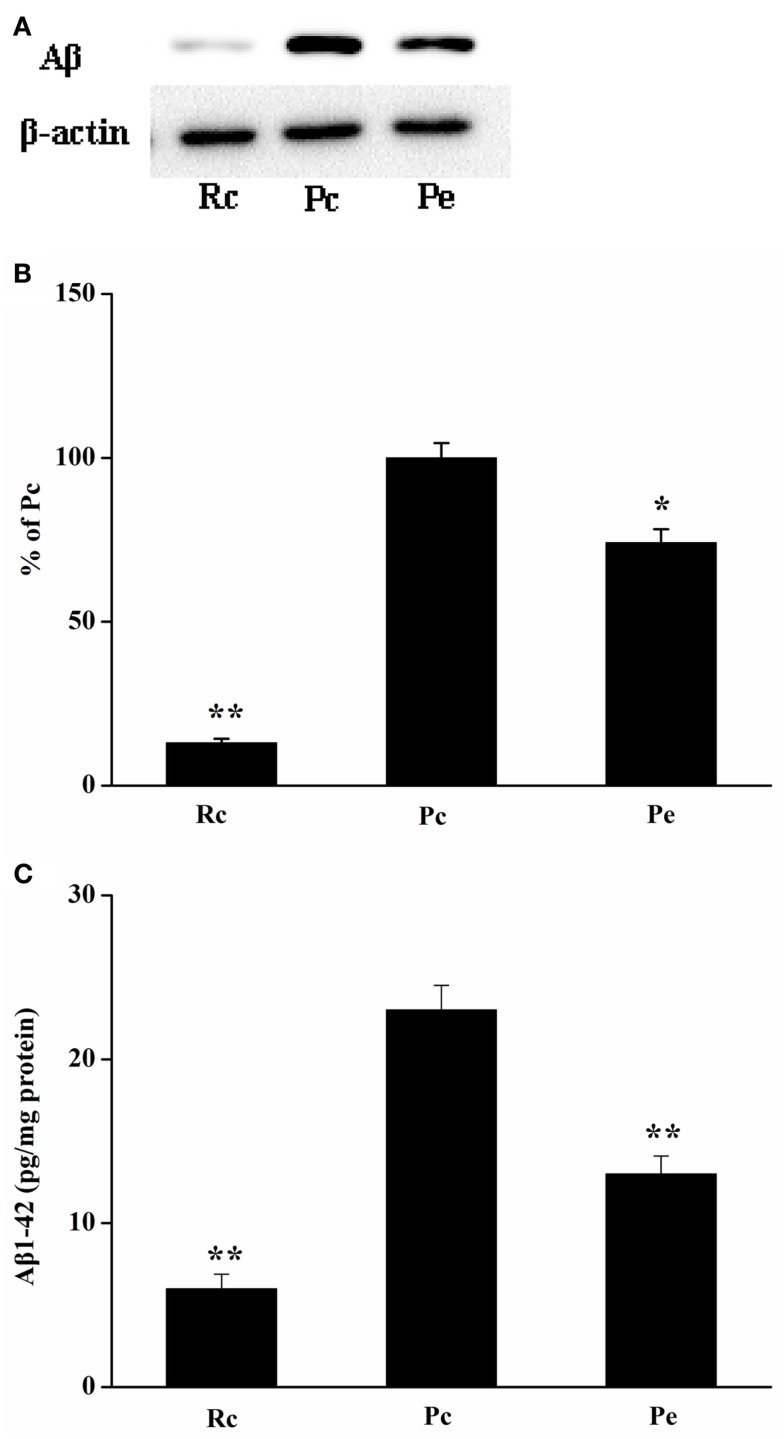
**Effects of EA administration on Aβ production in the SAMP8 mouse hippocampus**. **(A)** Representative western blotting analysis of Aβ. **(B)** Quantification of **(A)** based on densitometry showed that the protein levels of Aβ were markedly reduced in the Pe mice hippocampus compared with that of Pc mice hippocampus. β-actin was used as an internal control. **(C)** Results of ELISA Aβ1-42 assays showed that EA treatment resulted in a decrease in Aβ production. The level of Aβ1-42 was standardized against proteins obtained from hippocampus tissue and is expressed as Aβ1-42 pg/mg of tissue protein. *P < 0.05, **P < 0.01 versus Pc.

To investigate the effects of EA on Aβ production in the SAMP8 mouse brain, we used western blotting analysis for Aβ and ELISA for Aβ1-42. Western blotting analysis showed that Aβ expression was increased in the hippocampus of SAMP8 mice compared with that of the hippocampus of SAMR1 mice. Furthermore, EA treatment reduced Aβ expression (Figures 2A,B). In addition, we analyzed the levels of Aβ1-42 in the hippocampus using ELISA. Consistent with the results of western blotting analysis, increased Aβ1-42 levels were observed in the hippocampus in Pc mice compared with Rc mice. Moreover, Pe mice showed a significant reduction in the levels of Aβ1-42 compared with Pc mice (Figure 2C).

### EA inhibits BACE1 expression in the SAMP8 mouse hippocampus

**Figure 3:**
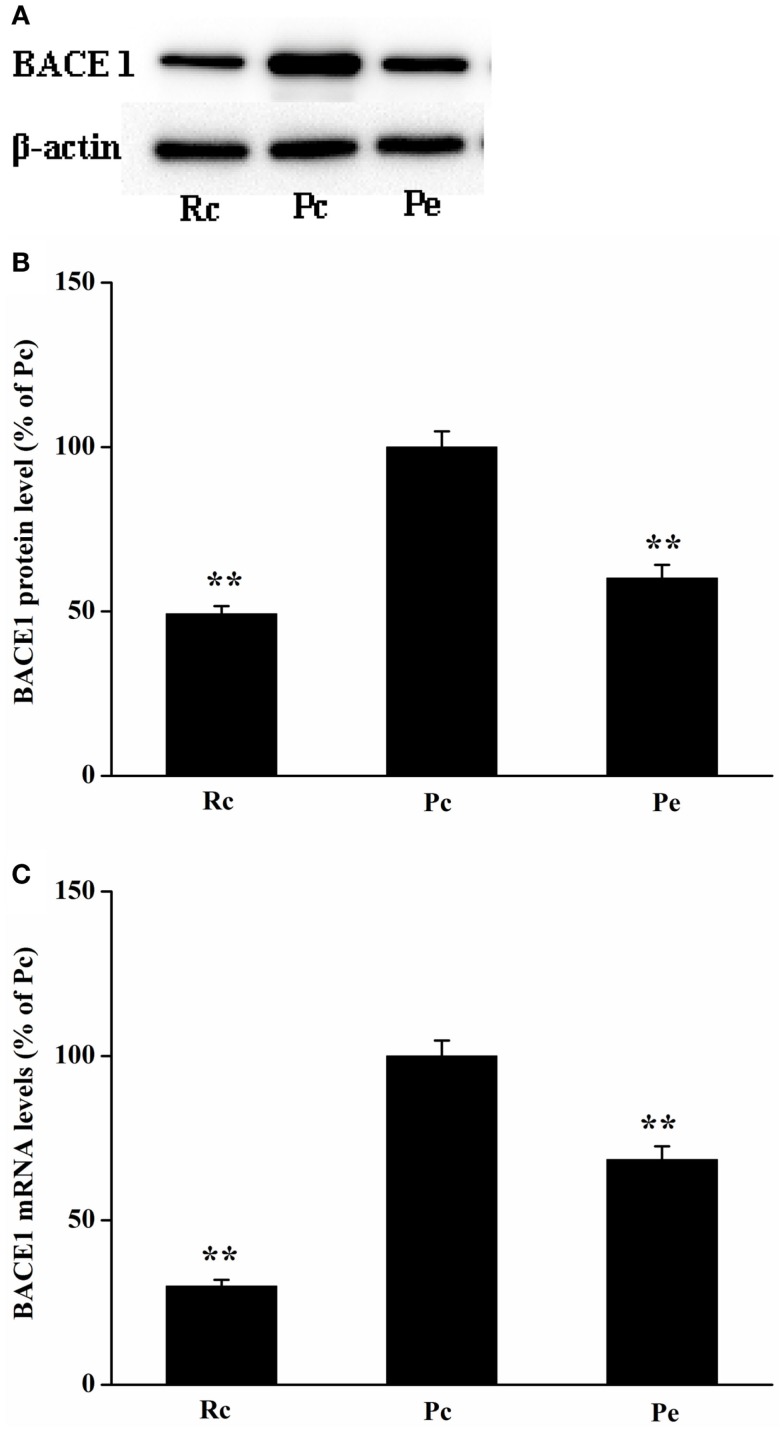
**Effects of EA treatment on BACE1 expression in SAMP8 mouse hippocampus**. **(A)** Representative western blotting analysis of BACE1. **(B)** Quantitative analysis of the protein expression of BACE1 showed that the level of BACE1 protein was reduced in Pe mice compared with that of Pc mice. **(C)** qRT-PCR was used to measure mRNA levels. BACE1 mRNA levels were normalized against GAPDH mRNA level and compared with Pc. *P < 0.05, **P < 0.01 versus Pc.

Because BACE1 is a key enzyme in Aβ generation, reduced levels of Aβ1-42 may result from decreased biogenesis of BACE1. Thus, we investigated the effects of EA treatment on BACE1 expression in SAMP8 mice. BACE1 protein was measured using western blotting analysis. We found that BACE1 expression was increased in Pc mice compared with that of Rc mice and that EA reduced BACE1 protein levels in SAMP8 mice (Figures 3A,B). To define whether changes in BACE1 protein expression were due to alterations in BACE1 transcription, we further examined BACE1 mRNA levels using quantitative real-time reverse transcription polymerase chain reaction (qRT-PCR). As expected, EA treatment resulted in a significant decrease in BACE1 mRNA levels compared to Pc (Figure 3C).

## Discussion

The present study demonstrated that EA treatment improves spatial memory in the SAMP8 mouse model of AD. Furthermore, we have shown that EA treatment reduces Aβ production and BACE1 expression in the hippocampus of SAMP8 mice. Our data support that EA is effective for the treatment of AD in animal models.

It has been reported that compared with age-matched SAMR1 mice, cognitive impairment of SAMP8 mice could be detected beginning at 5 months of age and was severe at 7 months (Kang et al., 2014). In addition, from as early as 6 months of age, SAMP8 mice show Aβ deposition in the hippocampus that increases in number and extent with age (Del Valle et al., 2010; Kang et al., 2014). The tau hyperphosphorylation is significantly increased in the hippocampus of SAMP8 mice from 7 months of age (Jiang et al., 2015b) compared with SAMR1 mice. Taken together, SAMP8 mice have developed AD-like behavior and pathological hallmarks at 7 months of age. Moreover, a number of studies used 7 or 7.5-month-old SAMP8 mice to investigate the effect of EA and drug treatment on AD (Tan et al., 2014; Jiang et al., 2015a). Thus, we used SAMP8 mice at 7 months of age in this study. Additionally, Kang et al. have systematically evaluated the pathologic morphology in the hippocampus of SAMR1 and SAMP8 mice using Nissl staining, immunohistochemical staining (anti-Aβ antibody), and Golgi staining at different time points (Kang et al., 2014). Previous studies have shown that acupuncture reduces neuron loss and increases cell proliferation in the hippocampus of SAMP8 mice (Cheng et al., 2008; Li et al., 2012). On the basis of these results, our study emphasizes the effect of EA on Aβ production and BACE1 expression in SAMP8 mice.

Amyloid plaques are one of the characteristic pathological hallmarks of AD, and the deposition of Aβ is the main cause of plaque production. Aβ accumulation in the brain results in a cascade of cellular changes in AD pathogenesis. Thus, Aβ reduction or clearance from the brain may be an important therapeutic strategy for prevention and treatment of AD (Jakob-Roetne and Jacobsen, 2009; Mao et al., 2012). It has been demonstrated Aβ lowering capacity of anti-Aβ antibodies in transgenic models of AD and in AD patients (Bohrmann et al., 2012; Ostrowitzki et al., 2012). To examine the mechanism of EA treatment in AD mice, we investigated the effects of EA on Aβ expression in the hippocampus of SAMP8 mice. These results exhibited a significant increase in Aβ expression in the hippocampus of 8-month-old SAMP8 mice, which was consistent with a previous study showing that SAMP8 mice demonstrate deposition of Aβ from 6 months of age (Del Valle et al., 2010). EA treatment obviously decreased Aβ expression. Because Aβ1-42 is more cytotoxic and is more likely to aggregate and form plaques than Aβ1-40 (Vattemi et al., 2009), we further examined Aβ1-42 levels using ELISA in our study. Our results supported that the levels of Aβ1-42 in the hippocampus were significantly increased in SAMP8 mice compared with SAMR1 mice (Li et al., 2009). In addition, EA treatment reduced the levels of Aβ1-42 in SAMP8 mice. Taken together, these results suggested that EA treatment might reduce Aβ production.

Aβ, the product of the large type1 trans-membrane protein APP, is produced in a two-step proteolytic process initiated by BACE1 and followed by γ-secretase. Thus, BACE1 expression levels are strongly correlated with Aβ levels. In addition, BACE1 overexpression increases Aβ formation in APP transgenic mice (Bodendorf et al., 2002), whereas BACE1 knockout mice produce little or no Aβ (Luo et al., 2001), indicating that BACE1 expression plays a critical role in Aβ biosynthesis. Thus, targeting BACE1 is the focus of AD research in the prevention of Aβ generation in AD (Sathya et al., 2012). It has been reported that several BACE1 inhibitors have entered clinical trials (May et al., 2011; Hamada and Kiso, 2013; Hilpert et al., 2013). However, a recent clinical trial with a BACE1 inhibitor had to be halted due to liver toxicity (Lahiri et al., 2014). EA has demonstrated therapeutic potential for the treatment of AD and has no adverse side effects. Our results showed that EA improves cognitive deficits in SAMP8 mice, which are consistent with previous studies (Cheng et al., 2008; Li et al., 2012, 2014; Wang et al., 2014a). In this study, we further examined the effects of EA on BACE1 expression in SAMP8 mice. The mRNA levels and protein levels of BACE1 are increased in the hippocampus of SAMP8 mice compared with that of the hippocampus of age-matched SAMR1 mice, which is consistent with a previous study (Orejana et al., 2015). Furthermore, our results showed that EA reduces BACE1 expression. It has been reported that targeting BACE1 with siRNAs reduced Aβ production and the neurodegenerative and behavioral deficits in an APP transgenic mouse model of AD (Singer et al., 2005). Lowering BACE1 expression reduced the formation of Aβ, thereby preventing its subsequent aggregation into toxic aggregates (Citron, 2002; McConlogue et al., 2007). In addition, triptolide and icariin, the major active components extracted from a traditional Chinese herb, have been reported to inhibit BACE1 expression, attenuate Aβ production and deposition, and improve cognitive deficits in transgenic mouse models of AD (Wang et al., 2014b; Zhang et al., 2014). Similar to our results, these results support the hypothesis that reduced BACE1 activity has great therapeutic potential for the treatment of AD, and BACE1 is a prime therapeutic target for lowering Aβ levels in AD.

We showed previously that EA activates AMPK expression and upregulates SIRT1-dependent PGC-1α expression in SAMP8 mice (Dong et al., 2015). Moreover, a recent study has shown that SIRT1-PPARγ- PGC-1 represses BACE1 transcription and BACE1 protein levels (Wang et al., 2013). In this study, we found that EA reduced BACE1 mRNA and protein levels and decreased Aβ levels. On the basis of these data, we proposed that EA upregulates SIRT1-PPARγ- PGC-1, which represses BACE1 expression, thereby reducing Aβ production and consequently improving cognitive deficits in an AD animal model. This may represent a potential mechanism underlying EA improvement of cognitive deficits in AD.

In summary, the current study demonstrated that EA treatment reduced Aβ production and BACE1 expression in the hippocampus of SAMP8 mice, thus improving learning-memory abilities. These novel findings suggest that EA treatment may have the potential to block or delay the pathological progression of AD.

## Conflict of Interest Statement

The authors declare that the research was conducted in the absence of any commercial or financial relationships that could be construed as a potential conflict of interest.

## Abbreviations

AD, Alzheimer’s disease; Aβ, amyloid β-peptide; BACE1, β-site amyloid precursor protein-cleaving enzyme 1; EA, electroacupuncture; ELISA, enzyme-linked immunosorbent assay; GAPDH, glyceraldehyde-3-phosphate dehydrogenase; MWM, Morris water maze; PGC-1α, peroxisome proliferator-activated receptor-γ co-activator-1α; qRT-PCR, quantitative real-time reverse transcription polymerase chain reaction; SAMP8, senescence-accelerated mouse prone 8; SAMR1, senescence-accelerated-resistant mouse 1; siRNA, small interfering RNA; SIRT1, Sirtuin 1.
